# Traditional Safe Triangle Approach Versus Kambin’s Triangle Approach: Does Approach Really Matter in Transforaminal Epidural Steroid Injection (TFESI) for Lumbar Disc Herniation?

**DOI:** 10.7759/cureus.48701

**Published:** 2023-11-12

**Authors:** Sandesh Agarawal, Manoj K Ramachandraiah

**Affiliations:** 1 Orthopaedics, Sri Devaraj Urs Medical College, Sri Devaraj Urs Academy of Higher Education and Research (SDUMC-SDUAHER), Kolar, IND

**Keywords:** lumbar disc herniation, safety triangle, numerical rating scale, oswestry disability score, : kambin's triangle

## Abstract

Background: Lumbar disc herniation (LDH) is one of the primary causes of back and leg pain affecting today’s working population and is a major contributor to sickness absenteeism, creating a substantial socio-economic burden. Low back pain is one of the leading causes of physical disability in both old and younger age groups. Low back pain has a point prevalence of 12%, a year-on-year prevalence of 38%, and a lifetime prevalence of 40%. The aging population leads to a rising number of individuals affected by lower back pain. The study aims to compare the outcome of percutaneous transformational epidural steroid injection (TFESI) for LDH using the traditional safe triangle approach versus Kambin’s triangle approach.

Materials and methods: A retrospective cohort hospital-based observational study was conducted among a total of 90 patients who had underwent percutaneous epidural steroid injection using the traditional safe triangle approach versus Kambin’s triangle approach for LDH. Patients were identified through electronic medical record (EMR) documentation submitted to the Department of Orthopaedics, R.L. Jalappa Hospital Centre, during the period of May 2022 to May 2023.

Results: The majority of patients in Group A belonged to the 61-65 years and in Group B to the 71-75 years, respectively. The overall success rate of the procedure was higher in the safe triangle approach, and there was an association found between the type of procedure and the successful rate. There was no statistically significant difference between the two approaches, namely, traditional safety triangle approach and Kambin’s triangle approach, in terms of Numerical Rating Scale (NRS) score and Oswestry Disability Index (ODI) score at pre-injection and at one month and three months post-injection.

Conclusion: The Kambin's triangle approach is just as effective for the interim outcome as the subpedicular approach and provides significant advantages. The Kambin's triangle approach may be used as an alternative to TFESI in situations where needle tip placement in the anterior epidural is challenging.

## Introduction

Low back pain is one of the leading causes of physical disability in both the old and younger age groups [1]. Low back pain has a point prevalence of 12%, a year-on-year prevalence of 38%, and a lifetime prevalence of 40% [2]. The aging population leads to a rising number of individuals affected by lower back pain. Lumbar disc herniation (LDH) is one of the primary causes of back and leg pain affecting today’s working population and is a major contributor to sickness absenteeism, creating a substantial socio-economic burden [3]. The condition affects an estimated 11% of patients presenting to their primary care provider worldwide, with an annual incidence estimated to be 22% [4,5].

Generally, the use of first-line conservative management strategies, such as analgesic medications, steroid injections, and physical therapy, usually results in symptomatic relief in over 90% of patients within 12 weeks of symptom onset [6]. The pathophysiology of radicular pain involves both mechanical nerve compression, mainly disc herniation, and inflammatory processes [7]. Treatment options have been considered by various expert-led guidelines, but no consensus exists due to a scarcity of class I evidence [8,9]. Generally, steroid nerve root injections are recommended as a nonoperative approach for failed lifestyle-modifying treatments and failed pharmacologically managed cases [10].

Transforaminal epidural steroid injections (TFESIs) are an effective nonsurgical treatment option for patients with radiculopathy in whom more conservative treatments are not effective and had a success rate of 84%[11]. These procedures are effective and safe in relieving pain. Indications, evidence, and safety considerations for the technique have been identified [12]. While there are many different approaches to TFESI, the subpedicular method is the most widely used [13]. The subpedicular approach has been associated with spinal cord infarcts that have occurred as a result of neurovascular injury [14]. While this is a rare but potentially life-threatening complication, it has been reported to occur on a small number of occasions. The Kambin's triangle approach is the most effective approach to TFESI and offers significant benefits in terms of neurovascular complications [15].

The current study aimed to investigate the outcomes of TFESI for LDH using the traditional safe triangle approach versus Kambin's triangle approach. The clinical outcomes were assessed in terms of Oswestry Disability Index (ODI) and Numerical Rating Scale (NRS) during the preoperative period and one-month and three-month postoperative periods.

## Materials and methods

This retrospective cohort hospital-based observational study included patients who had underwent percutaneous epidural steroid injection using the traditional safe triangle approach versus Kambin’s triangle approach for LDH. Patients were identified through electronic medical records submitted to the Department of Orthopaedics, R.L. Jalappa Hospital Centre, during the period of May 2022 to May 2023. Patients were allocated into two groups: Group A included patients receiving TFESI through the traditional safe triangle approach; Group B included patients receiving TFESI through Kambin’s triangle approach.

Data collection was performed after obtaining ethical approval from the Institutional Ethics Committee of Sri Devaraj Urs Medical College, Kolar, Karnataka, India (approval number: DMC/KLR/IEC/179/2023-24). Patients between 20 and 80 years of age presenting with unilateral L5 radiculopathy for more than three months and no symptom improvement or continuation of pain for more than one month after physical therapy and medications were included in the study. The patient with unilateral L5 radiculopathy for less than three months, history of previous surgery, infections, cancer, severe neurological deficits, cauda equina syndrome, uncontrolled medical or psychiatric illness, allergy to contrast medium, and steroids was excluded from the study.

The data collection was conducted using electronic medical records, which contain socio-demographic and clinical history as well as outcome details of the patients. The ODI and NRS scores were calculated pre-injection at one and three months post-injection, respectively. The data were put into an Excel sheet in Microsoft Office and analyzed using IBM SPSS Statistics for Windows, Version 24.0 (Released 2016; IBM Corp., Armonk, New York, United States). Scale variables with normal distribution are presented as mean (standard deviation, SD). Proportions are presented as a number (%). A univariate comparative analysis of scale variables with normal distribution was performed using an unpaired, two-tailed t-test. Categorical data were analyzed using the chi-square test. A p-value of <0.05 was considered statistically significant.

Methods of injection

*Traditional Safe Triangle Approach/Subpedicular Approach* 

Patients were positioned in a prone position, cushioned with pillows beneath the abdomen, to facilitate lumbar stenosis alleviation. The correct lumbar segment was determined based on the oblique perspective of the Scotty Dog Shadow. To align the C-arm, the lower endplate of the C-arm was adjusted and rotated 15-30° to approximate the Scotty Dog Shadow. After disinfecting the site, a 3.5-inch 22-gauge needle was moved towards the subjacent pedicle and the inferolateral interarticularis (Safe Triangle) for the superior intervertebral foramen. When the needle had reached the inferior border, the C arm was rotated to its lateral position. The incubation line was positioned at the bottom of the line, and the needle was rotated to the lateral view. The needle was then directed to the anterior area and the upper region of the intervertebral foramen. When the needle reached the last site, a blood aspiration test was conducted to confirm the blood detection. Subsequently, 1 cc of non-ion contrast agent was injected under real-time fluoroscopy to determine if the agent had been administered into the anterior section of the epidural space. The procedure was performed under the guidance of the same clinician; photographs were taken before and after the contrast agent was administered. Subsequently, a second injection of 2 cc of pre-filled lidocaine-controlled syringe (containing 1.5 mL + 20 mg triamcinolone) with 0.5% lidocaine was administered.

Kambin’s Triangle Approach

Patients were positioned in a prone position, cushioned with pillows beneath the abdomen, to facilitate lumbar stenosis alleviation. The X-ray projection was directed towards the epiphysis plate of the upper and lower vertebral bodies, while the C-arm angle was adjusted by 20-35° towards the region, allowing the upper articular process to be seen at the center of the intervertebral disc. A 3.5-inch 22-gauge spinal needle was then inserted into the skin, parallel to the X-ray projection path, touching, directing laterally, and advancing by 2-3 mm. The needle was then positioned in a medially aligned position at the 5 o'clock position of the upper pedicle in the antero-posterior view with no further progressions. This was followed by the needle being positioned in the postero-inferior region of the intervertebral foramen in the lateral view. Once the needle had been secured to the last site, a single injection of 1 cc of non-ion contrast agent was administered to measure the position and degree of contrast agent diffusion. Subsequently, a second injection of 2 cc of pre-filled lidocaine-controlled syringe (containing 1.5 mL + 20 mg triamcinolone) with 0.5% lidocaine was administered.

## Results

**Figure 1:**
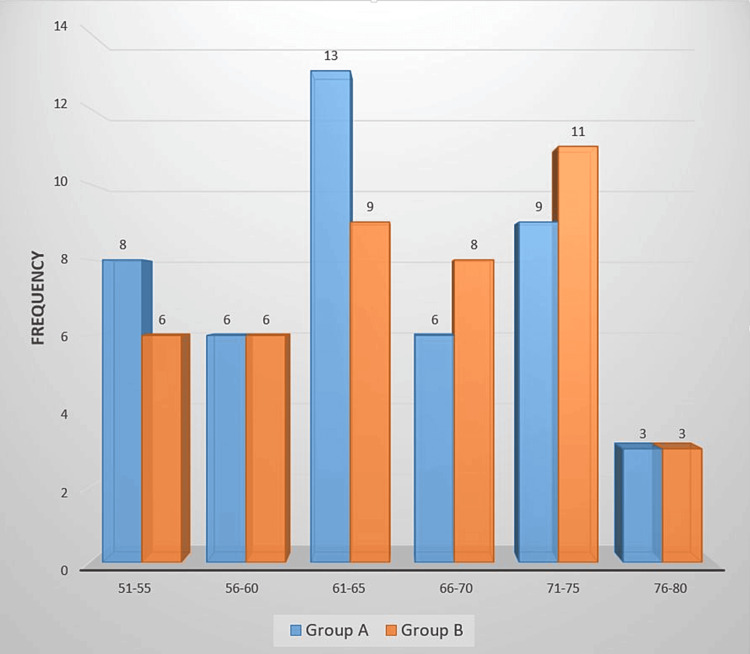
Age group-wise distribution among patients (n=90).

In Group A, the majority of patients were aged 61-65 years, while in Group B, the majority of participants were aged 71-75 years. Three participants in both groups were aged 76-80 years. The overall mean age of the study participants was 65.4 ± 8.13 years, as shown in Figure 1.

The mean BMI of Group A was 24.34 kg/m^2^ and that of Group B was 23.9 kg/m^2^. The mean duration of pain was 5.2 years and 4.63 years in Group A and Group B, respectively. There was a significant difference in mean age between the two groups. There was no statistically significant difference in mean BMI or pain duration between the two groups, as depicted in Table 1.

**Table 1:** Comparison of general characteristics of study participants of both groups (n=90).

Variable	Group A	Group B	Z test	p-value
Mean age (in years)	64.1 ± 7.5	65.9 ± 8.47	-2.95	0.003
Mean BMI (kg/m^2^)	24.34 ± 2.5	23.9 ± 3.1	0.723	0.469
Pain duration (in years)	5.2 ± 1.91	4.63 ± 2.04	0.936	0.348

**Figure 2:**
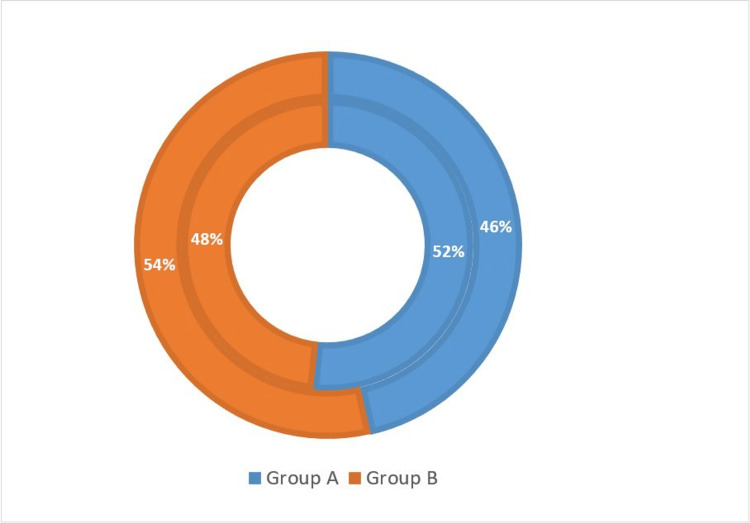
Gender-wise distribution (n=90).

In the graph, inner circle represents the females and outer circle represents the males. In Group A, 46% of participants were male and 52% female, while in Group B, 48% of participants were female and 54% male, as depicted in Figure 2.

A total of 34 patients in Group A and 23 patients in Group B underwent successful procedures. The overall success rate of the procedure was higher in the Safe Triangle approach compared to the Kambin’s Triangle approach for TFESI, and an association was observed between the type of procedure and the successful rate, as depicted in Table 2.

**Table 2:** Success rate among both groups.

Successful procedure	Group A	Group B	Chi-square test	p-value
Yes	34	23	5.78	0.0161
No	11	22		

**Figure 3:**
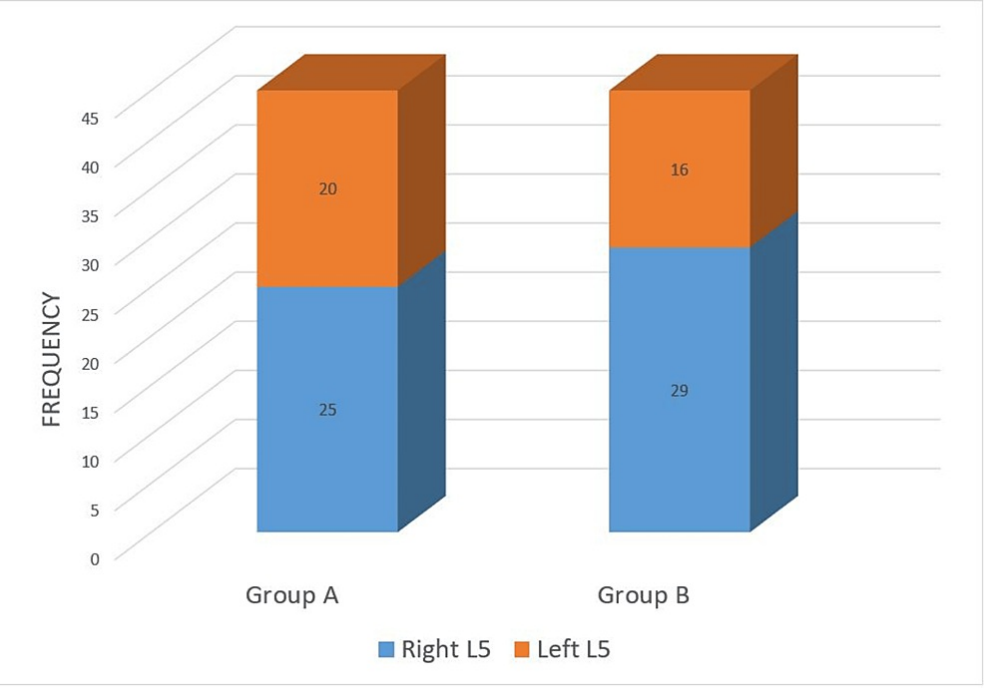
Target root for insertion among patients.

In 25 patients from Group A and in 29 patients from Group B, the right-sided L5 nerve root was targeted for insertion, as shown in Figure 3.

The mean pre-injection ODI score was 18.27 in Group A and 17.84 in Group B. The mean ODI score at three months of follow-up was 3.93 in Group A and 4.51 in Group B. There was no statistically significant difference in ODI score between the types of approach at pre-injection at one and three months post-injection, as depicted in Table 3.

**Table 3:** Comparison of ODI score among study participants. ODI: Oswestry Disability Index.

ODI score	Group A	Group B	Z-test	p-value
Pre-injection	18.27 ± 3.7	17.84 ± 4.2	0.706	0.479
After one month	10.78 ± 3.4	9.8 ± 4.5	1.61	0.107
After three months	3.93 ± 1.8	4.51 ± 2.5	0.956	0.340

The mean pre-injection NRS score was 5.96 in Group A and 6.24 in Group B. The mean NRS score at three months was 4.3 in Group A and 3.47 in Group B. There was no statistically significant difference in NRS score between the types of approach at pre-injection at one and three months of duration, as depicted in Table 4.

**Table 4:** Comparison of NRS score among study participants. NRS: Numerical Rating Scale

NRS score	Group A	Group B	Z-test	p-value
Pre-injection	5.96 ± 0.7	6.24 ± 0.9	1.41	0.645
After one month	4.98 ± 0.8	5.02 ± 0.6	0.654	0.941
After three month	4.3 ± 1.1	3.47 ± 0.72	1.36	0.172

**Figure 4:**
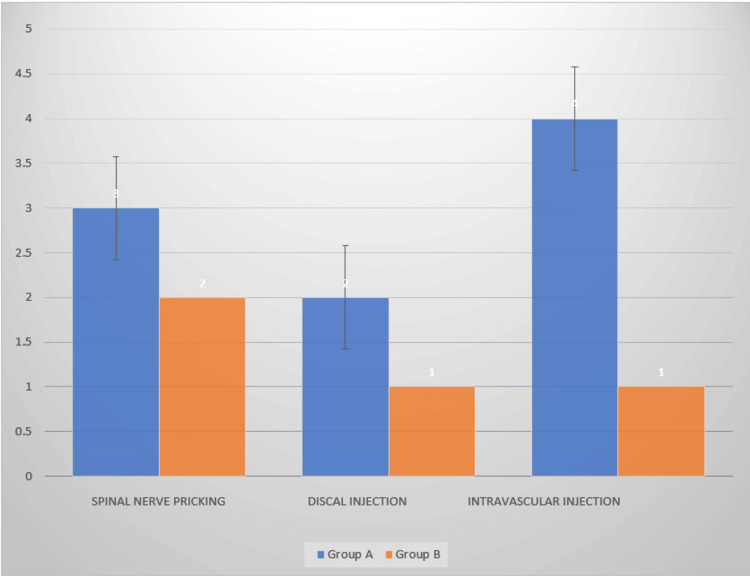
Complications among patients.

More complications were observed in Group A compared with Group B. A total of five patients had an inadvertent spinal nerve pricking, five had intravascular injection, and three additional patients had discal injection, as depicted in Figure 4.

There was no significant correlation found between type of procedure and ODI as well as NRS score, as depicted in Table 5.

**Table 5:** Correlation between type of procedure and ODI as well as NRS score. ODI: Oswestry Disability Index; NRS: Numerical Rating Scale. *3: Standard reference.

Variables		Odds ratio		p-value
		Group A	Group B	
ODI score	Pre-injection	1*	1*	-
	After one month	-1.21	2.9	0.543
	After three months	-1.34	2.46	0.196
NRS score	Pre-injection	1*	1*	-
	After one month	0.097	0.07	0.898
	After three months	1.1	0.34	0.644

Based on the patients' disability levels as measured by ODI, the mean pre-injection score was 18 for both groups. After three months of post-procedural recovery, the disability score ranged from 1 to 7 in Group A and from 1 to 10 in Group B, as depicted in Table 6.

**Table 6:** Patients' disability levels. ODI: Oswestry Disability Index; NRS: Numerical Rating Scale.

Variables		Group A	Group B
ODI score	Pre-injection	18 (10-14)	18 (10-14)
	After one month	10 (6-20)	11 (6-20)
	After three months	4 (1-7)	4 (1-10)
NRS score	Pre-injection	6 (4-7)	6 (4-9)
	After one month	5 (4-6)	5 (4-6)
	After three months	4 (1-5)	4 (1-5)

The range of pain score according to the NRS was 4-7 for Group A at pre-injection and 4-9 for Group B. The median score for three months after operative procedure was 4 for Group A and 4 for Group B, as depicted in Table 7.

**Table 7:** Patients' pain level. NRS: Numerical Rating Scale.

Variables		Group A	Group B
NRS score	Pre-injection	6 (4-7)	6 (4-9)
	After one month	5 (4-6)	5 (4-6)
	After three months	4 (1-5)	4 (1-5)

**Figure 5:**
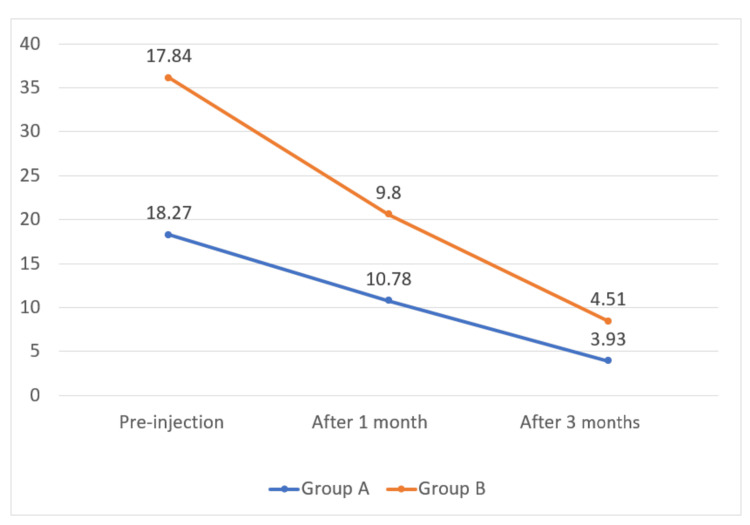
Mean ODI score on Y-axis. ODI: Oswestry Disability Index.

The mean ODI score in Group A was 18.27, 10.78, and 3.93, respectively, at pre-injection, after one month, and after three months. The mean ODI score in Group B was 17.84, 9.8, and 4.51, respectively, at pre-injection, after one month, and after three months post-injection, as depicted in Figure 5.

**Figure 6:**
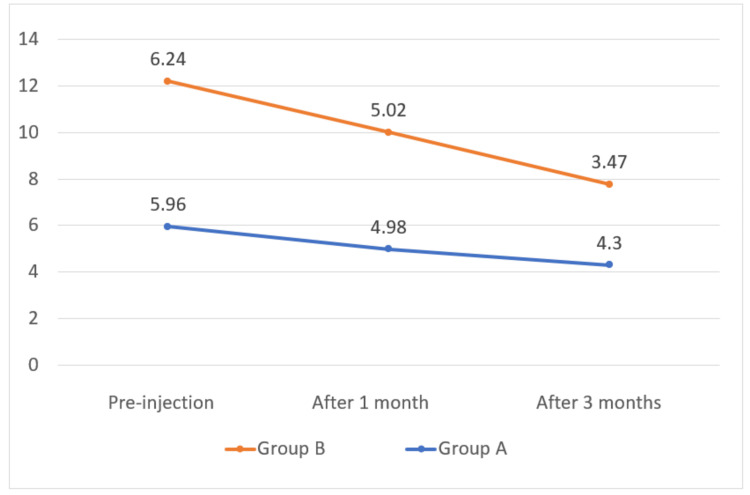
Mean NRS on Y-axis. NRS: Numerical Rating Scale.

The mean NRS score in Group A was 5.96, 4.98, and 4.3, respectively, at pre-injection, after one month, and after three months. The mean NRS score in Group B was 6.24, 5.02, and 3.47, respectively, at pre-injection, after one month, and after three months post-injection, as depicted in Figure 6.

## Discussion

Since inflammation has been shown to play a role in radicular pain development, corticosteroid therapy has become an effective treatment for inflammation and pain, with success rates ranging from 20% to 100% [16-19]. In 1971, Macnab pioneered the use of steroid injections to address inflamed nerve roots in the lumbar spine [16]. While there is a large body of literature on the results of transforaminal steroid injections for lumbar radiculopathy, the results are mixed. Manchikanti et al., in a systematic review of three approaches to steroid administration for the treatment of disc herniation (caudal, interlaminar, and transforaminal), found strong evidence for short-term (less than six months) pain relief and moderate evidence for long-term (more than six months) functional improvement [19]. Some other published studies and meta-analyses have described similar outcomes. However, the large variability between trials has resulted in different and often contradictory conclusions.

The traditional safe triangle in the lumbar spine is formed by the diagonal path of the nerve, base of the vertebral pedicle, and outer boundary of the vertebral body. This triangle is referred to as the "safe triangle" due to the fact that only the spinal nerves and blood vessels are located within this area [20]. This location is favored because agents can be injected into the anterior extradural space, i.e., the inflammatory site between the back of the herniated intervertebral disc and the anterior nerve root dural sleeve. The risk of damaging dura mater is decreased as the injection needle goes through the border of the lateral upper intervertebral foramen [21]. As the safe triangle is approached, caution should be exercised with regard to the Adamkiewicz artery (AKA). Although the AKA travels through the intervertebral foramen between T9 and L1 in 75% of cases, on rare occasions, it may pass through L2 and L4 [22]. The traditional safe triangle approach may cause blood vessel damage (AKA) or lead to complications (spinal cord infarction due to intravascular injections of particulate steroids) as the injection needle is inserted into the superolateral anterior part of the intervertebral foramen [23]. The first documented case of paralysis of the lower extremity due to ischemic spinal injury following a subpedicular injection of a steroid epidural in the lumbar, dorsal, and intervertebral foramen areas, despite the injection site being located in a safe triangle, was reported by Glaser and Falco [24,25]. In the current study, no such complications were noted.

Kambin’s triangle is a right triangle that is crossed over the dorsolateral disc. It is composed of the following components: the hypotenuse, which is the root of exit from the nerve root; the base, which is the width of the vertebra at the apex; and the height, which is the height of the dura/traversing nerve root [26]. Kambin's triangle approach for epidural injection minimizes the stimulation of nerve roots and tissue damage in the vicinity of the nerves, as well as the prevention of venous obstruction, nerve edema, and epidural hemorrhage [1]. This approach is considered safer than the traditional safe triangle approach. The only potential complication of this approach is the risk of injecting contrast medium directly into the disc, potentially resulting in diskitis. However, in this study, no such complications were observed [27].

Although Kambin’s triangle approach was thought to provide reduced therapeutic effects because the agent was injected into the lateral epidural space but not anterior, our study found no statistical difference. Similar results were observed in a retrospective study by Crall et al. [28] who reported that Kambin’s triangle approach did not show any difference or superiority in treatment effects when compared to the existing subpedicular approach [29,30]. Another study by Park et al. is also in agreement with the current study [31]. The Kambin’s triangle approach is as effective as traditional safe triangle approach in terms of functional outcome as analyzed by ODI and NRS scores. The results were in agreement with a similar study by Jeong et al. [30] who concluded that Kambin’s approach was superior to the subpedicular approach in treatment effects after four weeks in patients with LDH because the needle can be placed closer to the nerve root compressed by the herniated intervertebral disc.

The traditional safe triangle technique, commonly referred to as subpedicular, involves the insertion of a needle into the anterior section of the intervertebral foramen and traversing the nerve root. The spinal nerve root can be punctured during the injection procedure, as it is difficult for the needle to pass through the anterior epidural region through the secure triangle of severe spinal stenosis and sunken degenerative intervertebral discs. In contrast, in Kambin's triangle approach, the needle is inserted on the lateral view from the lower-posterior angle, thus reducing the risk of the spinal nerve roots being punctured.

The subpedicular approach is technically easier and places the drug in anterior epidural space. The injection needle progresses towards the safe triangle under the inferior surface of the pedicle to locate the superolateral spinal nerve involved, which is the inflammatory site between the back of the herniated intervertebral disc and the anterior nerve root dural sleeve. The risk of damaging the dura mater is minimized as the injection needle goes through the border of the lateral upper intervertebral foramen. Whereas due to the needle's close proximity to the AKA, which may enter the intervertebral foramen at L2-L4 level in 20% of healthy people, the subpedicular approach is likely to damage the blood vessel or trigger complications, such as spinal cord infarction, resulting in devastating outcomes.

Kambin's approach is safer and can protect the epidural and nervous system and prevent chronic nerve edema, epidural bleeding, and epidural scarring. This approach is less likely to cause irritation because the drug is injected at the posterior aspect of the intervertebral disc and enters the target nerve root slowly instead of going right into the stenosis region. It is hence preferred in severe stenosis and epidural fibrosis. But this approach has a high risk of injecting a contrast agent into the intervertebral disc because the drug is injected in close proximity to the intervertebral disc. There are chances of developing diskitis, which may impact the outcome of the injection. In this clinical trial, three cases of pricking of the spinal nerve root were observed in patients undergoing spinal stenosis with a severe decrease in intervertebral disc pressure under subpedicular administration, while two pricking events were observed under Kambin's triangle administration (p < 0.05) [26].

TFESI is a commonly performed procedure for treating lumbar disc herniation. Although various approaches are described in the literature, there are no studies to compare which is more advantageous and safer. Larger population prospective randomized control trials on these techniques need to be done to understand the safety and effectiveness of one over the other.

Limitations

The primary limitation of this study was the limited number of patients enrolled; thus, it was not possible to draw a definitive conclusion. The second issue was that the type and degree of disc herniation were not assessed on imaging, which may have an impact on the procedure's success rate. Additionally, the procedure's outcome may be affected by whether the primary source of pain is an intervertebral disk or a bony intervertebral foramen. Thirdly, there were several confounders, such as the duration of pain and functional status of the patient. We believe that the age distribution in our study had a significant effect on the results, e.g., ODI. In addition, the patients were only monitored for a period of three months following the procedure; thus, the short-term outcomes were only evaluated.

## Conclusions

LDH is the main cause of radiating pain due to mechanical compression and inflammatory responses. This condition may result in radiating pain in the lower extremities through narrowed intravertebral fusions due to the herniation of an intravertebral disc. This disc herniation occurs due to the degeneration and thickening of the ligamentum flavum, zygapophysial joint, and surrounding soft tissues. Nerve root pricking could be reduced during an epidural block using Kambin's triangle approach. This approach could potentially serve as a substitute for the subpedicular approach for the treatment of severe spinal stenosis, epidural fibrosis, and sunken degenerative intervertebral disc lesion, where it is difficult to insert the needle into the anterior area of the epidural space via the secure triangle. The Kambin's triangle approach is just as effective for interim outcome as the subpedicular approach and provides significant advantages. The advantage of Kambin’s triangle approach is needle placement anterior to epidural space in severe stenosis and reduced disc space, which is safe and does not injure any neurovascular structure. Also, the traditional safe triangle approach is a misnomer as it can damage the spinal cord due to an infarction. The Kambin's triangle approach may be used as an alternative to transformational epidural steroid injection in situations where needle tip placement in the anterior epidural is challenging.
